# Pancreatitis, Panniculitis, and Polyarthritis Syndrome as the First Symptom of Necrotizing Pancreatitis

**DOI:** 10.7759/cureus.55362

**Published:** 2024-03-01

**Authors:** Raagni Kumar, Kimberly Cichelli, Lawrence Brent

**Affiliations:** 1 Internal Medicine, Temple University Hospital, Philadelphia, USA; 2 Rheumatology, Temple University Hospital, Philadelphia, USA

**Keywords:** necrotizing pancreatitis, polyarthritis, erythema nodosum, pancreatitis, panniculitis

## Abstract

Pancreatitis, panniculitis, and polyarthritis (PPP) syndrome is a very rare cutaneous manifestation found in patients with acute pancreatitis. We report the case of a 44-year-old man presenting with erythematous, painful lesions on the lower extremities and ankle swelling. The rheumatology service was consulted for possible erythema nodosum. Extensive workup revealed elevated lipase and amylase levels, and computed tomography of the abdomen and pelvis revealed acute pancreatitis with necrotizing lesions and peripancreatic thoracic collections. There were also changes of chronic pancreatitis. The original skin manifestations were eventually identified as pancreatic panniculitis by skin biopsy. The patient was treated for pancreatitis and pleural effusions, and his skin and joint symptoms completely resolved. Pancreatic panniculitis with polyarthritis is rare but may be the first presenting symptom of pancreatic disease. Rheumatology may be consulted for these patients especially if there are only skin and joint manifestations and no abdominal pain. Misdiagnosis of pancreatitis can lead to poorer outcomes and delay in care. Therefore, pancreatic disease should be on the differential for any patient with panniculitis and polyarthritis.

## Introduction

Pancreatitis typically presents with digestive symptoms, especially nausea, vomiting, and abdominal pain; however, many extra-abdominal symptoms can also accompany the disease. One rare cutaneous manifestation is pancreatic panniculitis (PP) which occurs in 2-3% of individuals with pancreatic disease [1]. This condition consists of fat necrosis in the subcutaneous tissues, presenting as painful erythematous lesions, usually on the lower extremities [1,2]. Some patients can also develop joint swelling due to intraosseous necrosis, leading to a diagnosis of pancreatitis, panniculitis, and polyarthritis (PPP) syndrome. Interestingly, these symptoms can occur with minimal or no gastrointestinal manifestations [1-3]. The absence of gastrointestinal symptoms may lead to the misdiagnosis of pancreatitis and delay in care, resulting in poorer patient outcomes. We present the case of a patient with PP and polyarthritis as the first manifestations of necrotizing pancreatitis.

## Case presentation

We present the case of a 44-year-old Hispanic male with a past medical history of hypertension and alcohol and tobacco use who presented to the hospital for an acute-onset rash on his lower extremities for two days. The rash was initially described as circumferential, erythematous, raised, tender lesions that appeared below the knees without central clearing, drainage, or bleeding. The patient presented to the emergency department with pain and swelling in the left foot with difficulty with ambulation. He denied other joint pain, swelling, or prolonged morning stiffness. He endorsed associated subjective fever of one-day duration. He noted significant unintentional weight loss of about 100 lbs over the past year. He denied abdominal pain, nausea, vomiting, dyspnea, sputum production, or chest pain.

In the emergency department, he was found to be dyspneic with a dry cough. Laboratory results were notable for leukocytosis with a white blood cell count of 25.1 K/mm^3^ (reference range: 4-11 K/mm^3^) and an albumin of 1.9 g/dL (reference range: 3.5-5.9 g/dL). He was admitted to the hospital for further evaluation. 

**Figure 1:**
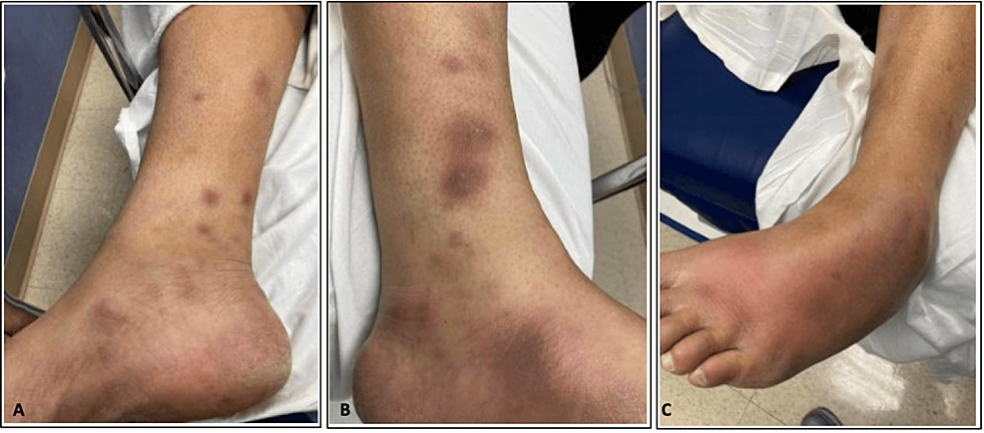
Erythematous nodules on right (A) and left (B) lower extremities. (C) Swelling of the left tarsometatarsal joints and left ankle joint.

Dermatology was consulted, and their findings were that this rash was consistent with a diagnosis of erythema nodosum. Rheumatology was then subsequently consulted the following day by which time the patient reported improvement in his rash. His exam was notable for raised, erythematous, non-tender nodules on both his lower extremities (Figure 1A, 1B) and significant swelling and tenderness of the left midfoot and ankle joints (Figure 1C).

**Figure 2:**
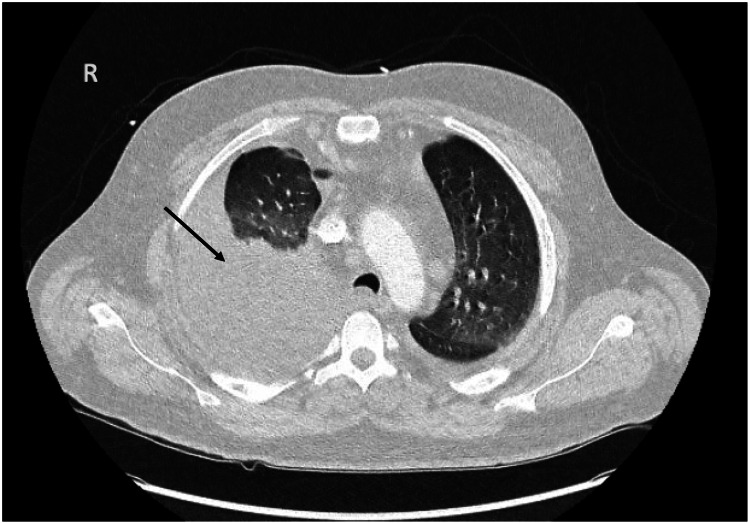
CT of the chest with a large right pleural effusion (arrow). CT: computed tomography

**Figure 3:**
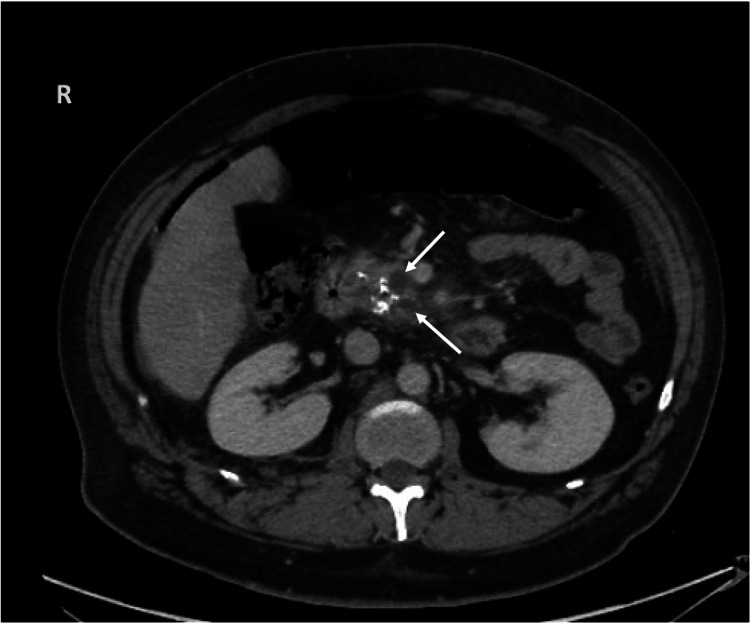
CT of the abdomen and pelvis with acute-on-chronic pancreatitis likely necrotizing with two cystic lesions/collections (arrows) within the pancreatic head and uncinate process. CT: computed tomography

**Figure 4:**
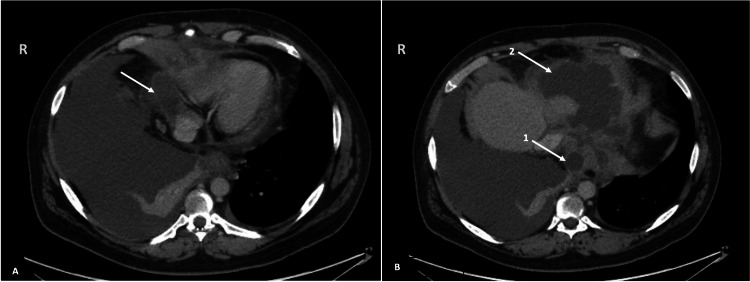
CT of the abdomen and pelvis showing a cardiophrenic collection which abuts the right heart (arrow) (A) and two thoracic fluid collections (B). The first collection is at the gastroesophageal junction and the diaphragmatic hiatus (arrow 1), and the second is a loculated gastrohepatic collection (arrow 2). CT: computed tomography

At that time, he continued to have dyspnea and was also complaining of diffuse tenderness to palpation of his epigastrium. Previously ordered laboratory tests including autoimmune and infectious disease testing were notable for a negative angiotensin-converting enzyme level, antinuclear antibody, anti-Ro antibody, anti-La antibody, anti-Smith antibody, antinuclear ribonucleoprotein antibody, hepatitis B serologies, human immunodeficiency virus, and antistreptolysin O titer. However, other results were significant for an amylase of 1,302 U/L (reference range: 0-105 U/L) and a lipase of 10,287 U/L (reference range: 73-393 U/L). A computed tomography (CT) scan of his chest showed a large right pleural effusion (Figure 2). A CT scan of the abdomen and pelvis showed acute-on-chronic pancreatitis, likely necrotizing (Figure 3) with multiple encapsulated peripancreatic collections, some extending into the thoracic cavity (Figure 4A, 4B). Fluid analysis of the right pleural effusion was consistent with exudative effusion with the presence of an elevated amylase of 42,178 U/L (reference range: 0-105 U/L).

**Figure 5:**
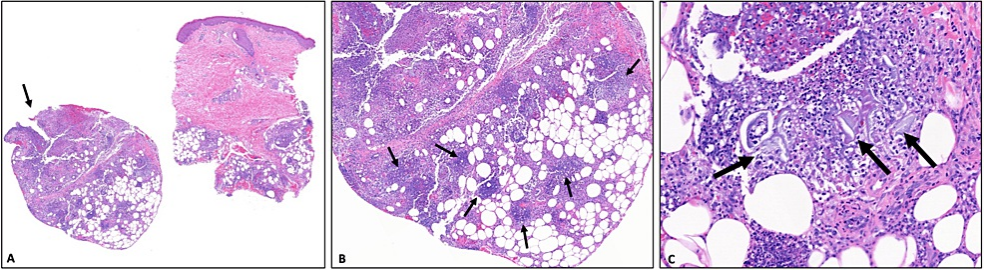
(A) A skin biopsy that shows acute lobular panniculitis with fragmentation of the subcutis indicative of loose friable tissue (arrow) (40× magnification). (B) There is a dense infiltrate of neutrophils within the lobules with acute necrosis of adipocytes and degenerative changes (arrows) (100×). (C) Higher-power view shows early changes of saponification: anucleate blue-gray amorphous material surrounded by numerous neutrophils (arrows) (400×).

The diagnosis of the rash was then updated to PP. Dermatology performed a biopsy that showed acute lobular panniculitis with dense infiltrates of neutrophils within the lobules and acute necrosis of adipocytes and degenerative changes; higher-power view showed early changes of saponification, anucleate blue-gray amorphous material surrounded by numerous neutrophils (Figure 5).

These findings were consistent with a diagnosis of PP. The patient had a prolonged hospital course with treatment for his pancreatitis, ultimately requiring a chest tube for drainage of his right pleural effusion and endoscopic retrograde cholangiopancreatography with stent placement of his proximal pancreatic duct. He was able to be discharged home in stable condition with close follow-up planned.

## Discussion

Cutaneous manifestations of pancreatitis are often present either preceding or concurrent with gastrointestinal symptoms. The most common and benign skin presentations include jaundice, pruritis, xanthomas, and hyperpigmentation. The more uncommon cutaneous signs, including PP, can help signal the diagnosis and prognosis of pancreatic disease [4]. While PP is more commonly seen in middle-aged men with a history of chronic alcohol use [5], it has also been documented in cases of pancreatic malignancies including acinar cell carcinoma, ductal adenocarcinoma, and neuroendocrine tumors as well as other pancreatic complications like pseudocyst, fistulas, and pancreas divisum [1,5,6]. Therefore, the identification of PP should prompt the screening of pancreatic malignancy.

The pathophysiology of PP is not well understood but is thought to be due to high serum levels of lipase and other pancreatic enzymes (amylase, trypsin) which cause vessel permeability and fat hydrolysis. The free fatty acids and glycerol that are produced accumulate leading to fat necrosis and subsequent inflammation [3,4]. However, there are likely other factors contributing to the development of PP, given that there are cases of patients with PP and normal lipase levels [1,4,6]. Some proposals include cytokines, specifically adipokines, and immune complex deposition, although more investigation is needed to confirm these findings [6].

Clinically, patients with PP will present with painful erythematous subcutaneous lesions usually on the lower extremities but can occur anywhere on the body. These lesions can occasionally ulcerate and drain brown, oily secretions [1,3,6]. Patients with PPP syndrome will also have joint swelling and acute arthritis in one or more small or large joints [2]. The subcutaneous nodules in PP and PPP are similar to other skin conditions seen in rheumatologic conditions, including erythema nodosum, lupus panniculitis, scleroderma panniculitis, dermatomyositis panniculitis, polyarteritis nodosum, and sarcoid-related panniculitis [1,2,4,7,8]. Combining the skin nodules with joint inflammation, it can be difficult to distinguish between a rheumatologic etiology and pancreatic etiology, especially if the patient does not initially present with abdominal symptoms [7,8].

Determination can be made with skin biopsy which must include subcutaneous fat and histological analysis, which will reveal lobular panniculitis without vasculitis, fat necrosis, calcium deposition, and "ghost cells": adipocytes that have lost their nuclei due to necrosis, unique to PP [1,2]. Conversely, erythema nodosum by skin biopsy would reveal septal panniculitis without vasculitis with a lymphohistiocytic predominance [8].

Treatment for PP and PPP includes treatment of the underlying pancreatitis and identification of complications. The resolution of the subcutaneous nodules and joint inflammation tend to occur as the inflammatory phase of the pancreatitis resolves [1]. Analgesia with non-steroidal anti-inflammatory drugs and corticosteroids can help symptomatically, but there is minimal evidence suggesting that they reduce the duration of the disease [1,3]. Fortunately, our patient's panniculitis and arthritis symptoms began to improve once his pancreatitis was identified and treated.

## Conclusions

Given the number of rheumatologic diseases that present with panniculitis, it is not unreasonable to consult the rheumatology service for a patient with a rash and joint swelling and minimal abdominal symptoms, even before considering pancreatitis as the etiology. PP and PPP are rare manifestations of pancreatitis with which not all clinicians may be familiar. As in the case of our patient, there is the chance of misdiagnosis and a delay in treatment if pancreatitis is not identified early. This case suggests that pancreatic etiology should be in the differential diagnosis for any patient with skin lesions suspicious for panniculitis and polyarthritis.
